# Nanoformulations in epilepsy therapy – a systematic review of emerging strategies in status epilepticus

**DOI:** 10.1007/s40199-025-00575-w

**Published:** 2025-09-18

**Authors:** Nuraziemah Ahmad, Mohmad Farooq Shaikh, Mohammed Tahir Ansari, Alina Arulsamy

**Affiliations:** 1 Neuropharmacology Research Laboratory, Jeffrey Cheah School of Medicine and Health Sciences, Monash University Malaysia, Bandar Sunway, Malaysia; 2 School of Dentistry and Medical Sciences, Charles Sturt University, Orange, NSW Australia; 3 School of Pharmacy, University of Nottingham Malaysia, Semenyih, Selangor Malaysia

**Keywords:** Epilepsy, Status epilepticus, Anti-Seizure medications, Nanoformulations

## Abstract

Status epilepticus (SE) presents a life-threatening medical emergency condition characterized by prolonged seizures, leading to severe neuronal damage, morbidity and mortality, if not promptly treated. Immediate intervention is crucial, yet current anti-seizure medications (ASM) face limitations such as adverse effects otherwise in need of invasive administration routes such as intravenous ASMs. Nanoparticle-based drug delivery systems offer a promising approach to overcoming these challenges by enhancing drug solubility, stability, and brain bioavailability. This systematic review examines recent advancements in nanoformulations of ASMs for SE management. Through a comprehensive search and analysis of the literature, 12 relevant studies were identified, highlighting various nanoparticle types, formulation methods, and pharmacokinetic parameters. ASM nanoparticles exhibit favourable characteristics for drug delivery, including small size, high surface area, and tuneable physicochemical properties. These properties enable efficient drug transport across biological barriers, precise targeting, and controlled release, ultimately improving drug efficacy and reducing side effects. With an optimized release profile and non-invasive route such as intranasal administration, nano-formulated ASMs offer promising immediate therapeutic strategy for SE therapy. However, further research is warranted to optimize nano-formulation parameters, assess long-term safety, and validate clinical efficacy. Overall, nanoparticle-based ASM delivery holds great potential for revolutionizing SE treatment, offering hope for improved patient outcomes and quality of life.

**Graphical abstract:**

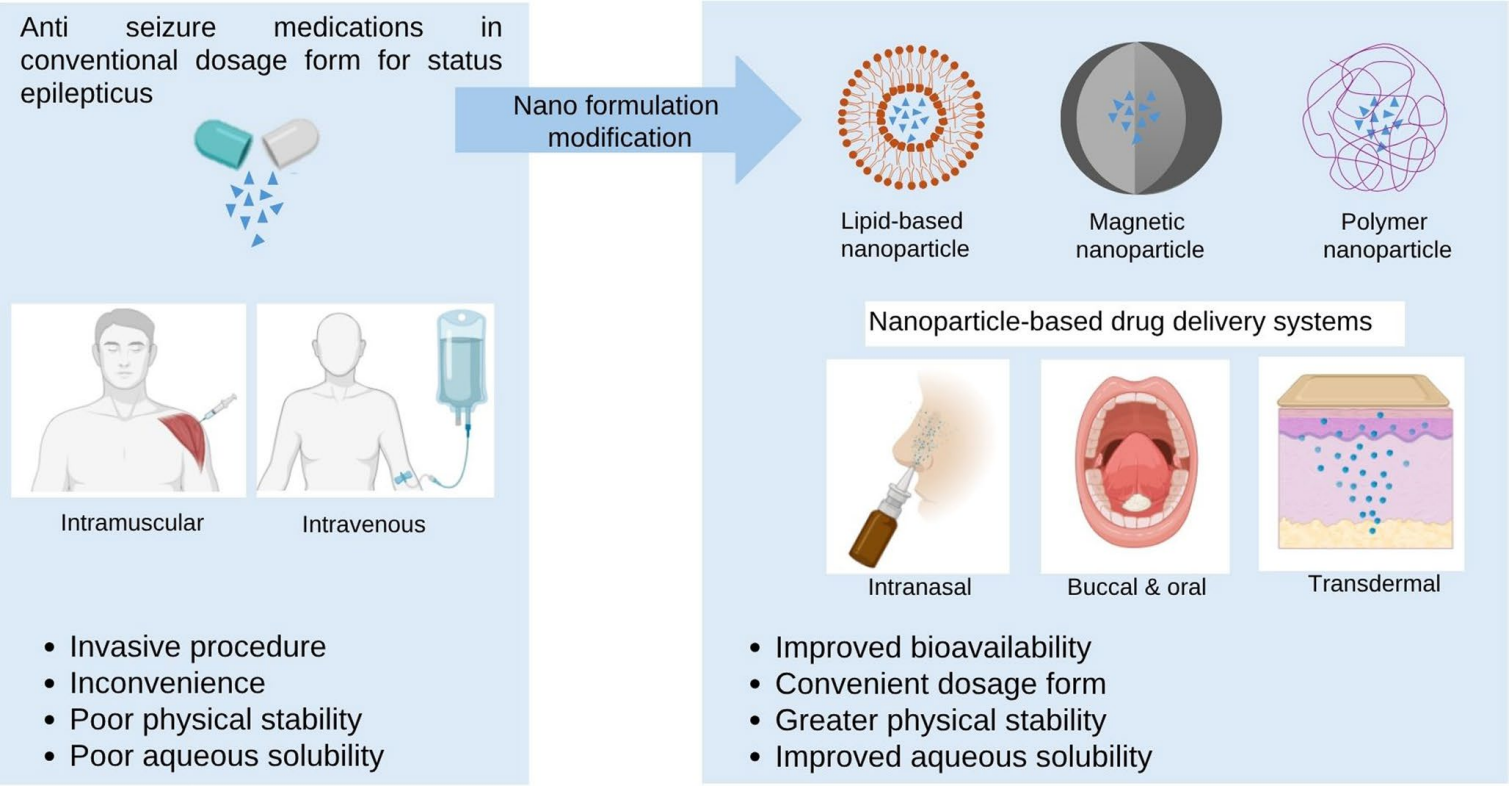

## Introduction

Status epilepticus (SE) is a life-threatening condition characterized by prolonged or repeated seizures without full recovery between them, resulting from excessive, abnormal, or synchronous neuronal activity in the brain. SE is considered a medical emergency.

During SE, a cascade of biochemical responses is triggered, leading to a chain of events that causes cellular damage, disruption, and potentially cell death. The sustained metabolic demands of rapidly firing neurons exacerbate the risk of significant morbidity and mortality, particularly when seizures persist beyond five minutes [1]. Early intervention is essential to prevent cerebral auto-regulatory failure and mitigate neuronal damage. However, despite the urgency of immediate intervention, the optimal treatment remains constrained. The current standard first-line therapy involving intravenous or rectal administration of anti-seizure medications (ASMs), often requires hospitalization and invasive procedures underscoring the urgent need for safer and efficacious treatment strategy for managing SE.

Benzodiazepines, a class of fast-acting ASMs, are most commonly used for the treatment of acute seizures and SE. They work by binding to the benzodiazepine-γ-aminobutyric acid (GABA) receptors, mediating the influx of chloride ions into the neurons and reducing the chances of action potential generation. Phenytoin is also an effective ASMs for the management of SE as well as chronic epilepsy, particularly for the treatment of partial and secondarily generalized seizures.

Despite extensive clinical trials, the current ASMs possesses several limitations including adverse drug effects. Particularly prolonged use of ASMs may lead to withdrawal symptoms, drug-drug interactions and economic burden, specifically in developing countries [2]. Chronic use of benzodiazepines can impair cognitive functions and can cause significant drowsiness, which can affect daily activities such as driving and thereby increasing the risk of accidents in patients.

Achieving control of uncontrolled seizures in SE is further complicated by the issue of drug resistance. Studies report that 20–30% of individuals suffer from drug-resistant epilepsy and a conclusive understanding of its mechanism still remains elusive. However, one of the main hypotheses suggests that the permeability of the blood-brain barrier (BBB) plays a crucial role in determining drug bioavailability and therefore speed of the treatment. Two main theories explain ASMs resistance: the target theory, which asserts that molecular targets of ASMs undergoes modifications thereby reducing the therapeutic efficacy, and the transporter theory, which suggests that an abnormal multidrug transporter activity leads to decreased effective concentration of ASM in brain [3].

Nanoparticles may offer a promising solution to overcome the BBB and achieve effective therapeutic ASMs concentrations in brain. These nanoparticles can traverse the BBB, by various mechanisms, including active transport through endocytosis or penetration through transporters and passive diffusion through the endothelial cells. Moreover, nanoparticles can enhance ASMs concentrations within BBB-forming epithelial cells or at the luminal surface, facilitating increased ASM penetration into the brain [4, 5].

Nanoformulation is highly capable of enhancing the bioactivity of drugs and altering their physicochemical characteristics. These nanoplatforms offers numerous advantages, particularly improving drug delivery to the brain, improving the drug solubility and stability, and modulating the release profiles. Such advancements are indispensable in the development of new pharmaceutical therapies [6]. Due to their small size (1–100 nm), high surface-to-mass ratio, favorable shape and structure, and distinct physical and chemical properties, the nanoparticles can easily penetrate cells, tissues, and biological barriers such as BBB [7]. Researchers are increasingly exploring these characteristics to optimize the efficacy and efficiency of current anti-seizure medications (ASMs) and leveraging nanoparticle versatility to address the challenges of drug delivery in neurological disorders.

The aim of this systematic review is to comprehensively evaluate the current available literature on nanoformulation modification of ASMs and elucidate their therapeutic potential for the management of SE. This review explores the relationship between different types of nanosystems, their physicochemical properties, and the pharmacokinetics of the nano-formulated ASMs, while assessing their efficiency and efficacy in the treatment of seizures and SE. This review will provide insights on enhancing the therapeutic outcomes of SE management through nanoformulation treatment strategies.

## Methodology

### Literature search and selection

**Fig. 1:**
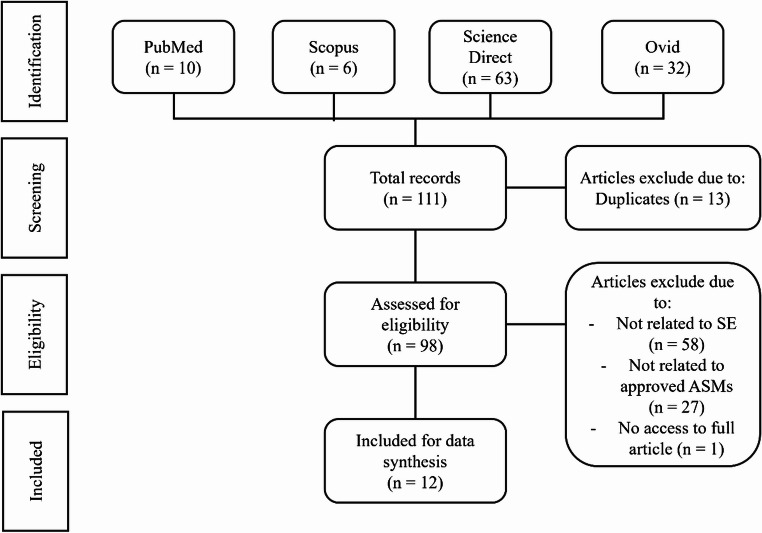
PRISMA flowchart on the selection process of literature articles

A systematic literature search was performed on four databases, including Google Scholar, PubMed, Scopus, and Science Direct, using the search terms [nano particle OR nanoparticle OR nanoformulation OR nano formulations] AND [status epilepticus]. The results were limited to articles published between January 2010 to Jan 2024. The literature search results were screened according to the inclusion and exclusion criteria. The inclusion criteria were original full-text research articles in the English language and articles that investigated nanoformulations in the context of SE. The exclusion criteria were articles labelled as review articles, conference papers, commentaries, book chapters or editorials, and lack of relevance to the aim of this manuscript whereby the articles excluded did not explore nanoformulations in the context of SE treatment (Fig. 1). All the literature search procedures, article selection, and data synthesis were performed according to the guidelines outlined by the PRISMA protocol [8].

## Data synthesis and the variables

Informative data describing the collected studies, including the author’s name, publication date, and the type of nanoparticle formulation, were extracted and summarised from the selected articles in Table 1. Additionally, formulation method, type of drugs, particle size and charges, entrapment efficiency, route of administration, efficiency, and nanoparticle stability were extracted and qualitatively described as well.

**Table 1:** Summary of selected article findings: formulation type, related problems with current drugs, physicochemical properties, drug release profile, and stability test

Formulation type	Drug	Problems addressed	Excipients	Physicochemical characterisation		Animal types	Drug release pattern	Stability	Formulation method	Reference
				Particle size (d. nm)	Zeta potential (mV)					
Transdermal administration										
Niosomes transdermal patch	Midazolam	Frequent dosing and low bioavailability	Span60, soybean oil, cholesterol	116.1 ± 84.5	+ 8.56 ± 1.2	ND	Initial burst followed by sustained release	90 days in different atmospheric conditions	Thin film hydration method	Shefrin et al. 2019 [10]
Intranasal administration										
Polymeric nanoparticle suspension	Lorazepam	Parenteral route: poor physical stability Oral & rectal route: slow onset, drug degradation, low compliance	PLGA, Poloxamer, acetone	167–318	−18.4	Sprague-Dawley rats	Initial burst with prolonged release (Korsmeyer-Peppas model)	90 days in accelerated temperature and humidity	Melt emulsification	Sharma et al. 2014[11]
Nano lipid carrier	Phenytoin sodium	Limited dosage form, unpredictable non-linear pharmacokinetic profile	Cholesterol, oleic acid, poloxamer 188	< 50	ND	Female Wistar rats	Immediate drug release	ND	Emulsification solvent evaporation	Nair, Vinayan and Mangalathillam 2021[12]
Polymeric nanoparticles	Clonazepam	Oral & parenteral route: insufficient quantity at the targeted site oral route: extensive first-pass metabolism, non-feasible	PLGA, Poloxamer 188	165.1	−14.8	BAL/c mice and New Zealand rabbits	Initial burst with prolonged release	ND	Ionic gelation method	Shah et al. 2021[13]
Chitosan loaded nanoparticles	Midazolam	Limited dosage form, high rate of first-pass metabolism	Chitosan, TPP	241.2 ± 12.3	ND	Albino Wistar rats	Initial burst with prolonged release	ND	Hydrotrope method	Shrestha et al. 2020[14]
Liquid crystalline nanoparticle	Rosuvastatin	Low aqueous solubility, poor oral bioavailability, high first-pass metabolism, poor permeation via BBB	Glyceryl monooleate, Poloxamer 407	219.2 ± 8.1		Swiss albino male mice	Biphasic following Korsmeyer-Peppas kinetics (Fickian diffusion)	ND	Nanoprecipitation	Ahmed et al. 2020[15]
PLGA loaded nanoparticle	Diazepam	Poor solubility Parenteral route: usage of cosolvents caused discomfort, adverse reaction Rectal route: variable bioavailability. Slow onset of action, low compliance	PLGA, poloxamer 407, acetone	148–337	(−15) – (−29.24)	Sprague-Dawley rats	Initial burst with prolonged release	18 months	2-step method; magnetic nanoaggregates synthesis and drug-loading	Sharma et al. 2015[16]
Magnetic nanoparticles	Phenobarbital	Intravenous route: slow administration, need access to professional medical care provider	Pluronic F-68, beta-cyclodextrin, ammonium hydroxide, ferrous sulphate heptahydrate	581	ND	Male Wistar rats	Immediate release	ND	Aqueous microtitration	Hemmat et al. 2023[17]
Nanoemulsion	Letrozole	Poor solubility, peripheral side effects	Triacetin, Tween 80, PEG-400	95.3 ± 2.3	(− 7.12) ± 0.12	Swiss albino male mice	Prolonged drug release	ND	Self-micro emulsifying drug delivery system (SMEDDS)	Iqbal et al. 2019[18]
Lipid-based nanosystem	Perampanel	poor solubility Parenteral route & nasogastric delivery: invasive, need specialized assistance	Migylol 812, Kolliphor RH40, Transcutol HP	20.07 ± 0.03	ND	Male CD-1 mice	Initial burst with prolonged release	Two weeks at room temperature without light	Ultrasonic technique	Meirinho et al. 2022[19]
Buccal & oral administration										
Nanosuspension powder	Midazolam	Poor solubility Parenteral & rectal route: less favorable in children Intranasal route: may cause sneezing and drug expulsion oral route: low bioavailability	Tween 80, Poloxamer (TP), 3-methyl chitosan (TMC)	197 ± 7	+ 31 ± 4	Rabbits	Initial rapid release followed by prolonged release	Three months	Solvent evaporation technique	Soroushnia et al. 2021[20]
Nanoparticles	Phenytoin and berberine	Low aqueous solubility	HPMC, PEG 6000, sodium caprate	321 (phenytoin) 471 (berberine)	−2.61 (phenytoin) −35.9 (berberine)	Wistar albino rats	Slow released	ND		Karthikeyini Senthilvel, Karuppaiyan and Mohammed Jamal Moideen 2019[21]

*ND *not determined, *PLGA *poly (lactic-co-glycolic acid), *PEG *polyethylene glycol, *TPP *triphenyl phosphate, *HPMC *hydroxypropyl methylcellulose

## SYRCLE RoB tool for bias assessment

Potential bias in the selected literature was assessed using the Systematic Review Center for Laboratory Animal Experimentation Risk of Bias (SYRCLE-RoB) tool. This tool was utilized to ascertain the quality of studies and evaluate bias in articles involving animal experiments. Ten parameters within the tool were considered, addressing six distinct types of bias: selection bias, performance bias, detection bias, attrition bias, reporting bias, and other potential biases. Comprehensive details regarding each bias category have been delineated elsewhere [9]. The categorization of biases involved recording each classification as either “yes” [+], “no” [-], or “unclear” [u].

## Results

The literature search resulted in 111 articles; 10 from PubMed, 6 from Scopus, 63 from Science Direct, and 32 from Ovid. Thirteen articles were excluded due to duplication. The remaining 98 literature articles went through a full-text assessment where the texts were searched for the main objectives of each study, types of active pharmaceutical ingredients (APIs), types of nanoformulation used, and the primary relevant study outcomes for comparison. Eighty-five articles were removed due to irrelevance to this systematic review aim, and one article was removed as the full-text was not available. Thus, 12 literature articles remained and were included in this review **(**Fig. 1**)**. The 12 literature articles have been summarised in Table 1.

The results presented in Table 1, highlight the optimized formulation from each study, reflecting the most favourable and optimised outcome. Among the selected studies, six studies focussed on the nanoformulations of benzodiazepine, while the rest explored nanoformulations of hydantoin, alkaloids, statin, aromatase, barbiturates, and α-amino-3-hydroxy-5-methyl-4-isoxazolepropionic acid (AMPA) antagonists. More than half of the experimental studies chose intranasal drug delivery, while others used oral, buccal, and skin routes of administration. The nanoparticle formulations predominantly used polymer or lipid carriers, with the lipid-based carrier displaying smaller particle sizes (20–219 d.nm) compared to the polymer-based carriers (148–241 d.nm). One study reported magnetic nanoparticles, which had a larger particle size of 581 d.nm. Zeta potential measurements were reported in seven studies, while in vivo studies were reported in eleven studies. The stability testing of nanoformulation were performed in five studies.

The experimental evidence of each study includes a control group, which uses the unmodified drugs which consistently exhibited less favourable outcomes compared to the nanoformulated drugs. Midazolam-loaded liposomal transdermal patches showed superior steady-state flux value and higher permeation rates compared to a midazolam transdermal patches and solutions [12]. Similarly, lorazepam-loaded PLGA nanoparticles exhibited an improved sustained release profile, enhanced uptake, and a superior brain activity compared to the lorazepam solution [13]. Another study emphasized the roles of phenytoin sodium loaded nano-lipid carrier for intranasal delivery, the study showed higher permeation rate and increased drug concentration in the brain within 5 min of administration compared to commercial intranasal midazolam while maintaining a comparable distribution in peripheral tissues [14]. Soroushnia et al. [15] also highlighted the importance of particle size for the absorption of the drugs through the buccal route. The optimized midazolam nano-suspension, characterized by smaller and uniform particles, demonstrating higher solubility, enhanced absorption and improved bioavailability compared to pure coarse midazolam powder drug. Another study reported nasal drug delivery of nanoformulation exhibited a seven- to eight-fold increase in drug releases compared to control groups, with significantly improved blood/brain ratios and drug accumulation in the brain region at every event of time [16]. Karthikeyani et al. reported a combination therapy with phenytoin and berberine nanoparticles administered as capsules showed an improved anticonvulsant efficacy [17]. It was found to be more efficacious than the free drug administration. Rosuvastatin (ROS) formulated into liquid crystalline nanoparticles demonstrated improved solubility and bioavailability. The nanoformulation provided substantial protection against pentylenetetrazole (PTZ)-induced SE, myoclonic jerk, and generalized seizure, as well as delaying their onset compared to control (intranasal ROS solution) [18]. Studies investigated by Sharma et al. [19] and Meirinho et al. [20] focused on the formulation of diazepam and perampenal into nanoformulation for delivery via intranasal, respectively. Optimized formulations for both drugs have also been shown to have improved targeting and better bio-distribution compared to each of their controls (diazepam and perampenal, respectively) with no sign of toxicity. Letrozole-loaded nanoemulsion (LET-NE) formulated by Iqbal et al. [21] offered enhanced hippocampal protection against kainic acid-induced neurotoxicity, showing superior anticonvulsant and neuroprotective effects compared to pure letrozole.

Table 2 represents the SYRCLE RoB tool’s bias assessment to analyse any bias in animal studies. Sequence generation was reported by one study (9%), while 91% of studies provided insufficient information. It was observed that 91% and 18% of the studies have a low-risk bias in baseline characterisation and random housing allocation, respectively. However, the evaluation of random sequence generation, allocation assessment, and blinded treatment could not be adequately assessed due to a lack detailed information across all studies. Random outcome assessment was conducted for 36% of the studies, while the rest of studies have unclear data. Only one study (9%) failed to provide complete outcome data, and the evaluation of outcome reporting in an additional 18% of the studies was incomplete due to in adequate information. All of the studies do not have selective outcome reporting and other sources of bias.

**Table 2:** Bias assessment using SYRCLE RoB tool where “yes” [+] depicts a low-risk bias, “no” [-] indicates a high-risk bias, and “unclear” [u] defines insufficient data to assess the risk of bias properly

	Sequence generation (selection bias)	Baseline characteristics (selection bias)	Allocation concealment (selection bias)	Random housing (performance bias)	Blinding (performance bias)	Random outcome assessment (detection bias)	Blinding (detection bias)	Incomplete outcome data (attrition bias)	Selective outcome reporting (reporting bias)	Other sources of bias (other)
Iqbal et al. 2019[18]	u	+	u	u	u	+	u	-	+	+
Nair et al. 2021[12]	u	+	u	u	u	u	u	u	+	+
Shrestha et al. 2020[14]	u	u	u	+	u	u	u	+	+	+
Soroushnia et al. 2021[20]	u	+	u	u	u	+	u	+	+	+
Shah et al. 2021[13]	+	+	u	u	u	+	u	+	+	+
Sharma et al. 2015[16]	u	+	u	+	u	u	u	+	+	+
Meirinho et al. 2022[19]	u	+	u	u	u	+	u	+	+	+
Karthikeyini et al. 2019[21]	u	+	u	u	u	u	u	+	+	+
Hemmat et al. 2023[17]	u	+	u	u	u	u	u	u	+	+
Sharma et al. 2014[11]	u	+	u	u	u	u	u	u	+	+
Ahmed et al. 2020[15]	u	+	u	u	u	u	u	+	+	+

A diverse range of formulations, particularly focusing on benzodiazepines and other ASMs like hydantoin and statins were analysed. A significant number of studies favoured intranasal delivery, which is critical for rapid seizure management, while highlighting the use of polymer and lipid carriers that influenced particle size and pharmacokinetic properties. These findings consistently demonstrated that nanoformulated drugs outperformed their unmodified counterparts in terms of bioavailability, brain uptake, and overall therapeutic efficacy, thereby supporting the review's aim to explore how these advanced formulations can enhance treatment outcomes for seizures. Additionally, the assessment of potential biases in the included studies underscores the need for rigorous methodologies in future research.

## Discussion

This systematic review has dissected a significant number of studies demonstrating the capability of nanoparticles or nanoformulation of ASMs as a ground-breaking approach in SE therapy. The nanoscale dimensions of these carriers contribute to their unique capabilities, allowing for the encapsulation of ASMs and facilitating targeted drug delivery.

### Nanoformulations in overcoming problems with ASMs

Many ASMs face challenges related to poor water solubility, a limitation that can impede their absorption and therapeutic efficacy [10]. Benzodiazepines, in particular are known to be lipophilic and exhibit poor water solubility at physiological pH, as addressed in Table 1. To address this, current formulations heavily rely on the use of organic solvents, cosolvents, and surfactants to enhance the solubility of benzodiazepines. Even the recently FDA-approved intranasal benzodiazepines Nayzilam and Voltaco, contains relatively high concentrations of organic cosolvents and surfactants, raising concerns over potential irritation to the nasal lining and long-term toxicity towards peripheral cells [11]. Nanoformulations address these issues by encapsulating these hydrophobic drugs, augmenting their solubility and bioavailability. Furthermore, the transformation of ASMs into nanoformulations provides an opportunity to incorporate excipients, such as solubility enhancers, permeability enhancers, and emulsifying agents. These additional facets allow for the modulation of the chemical properties of ASMs, contributing to an improved formulation that aligns with the specific requirements of effective seizure management

**Fig. 2:**
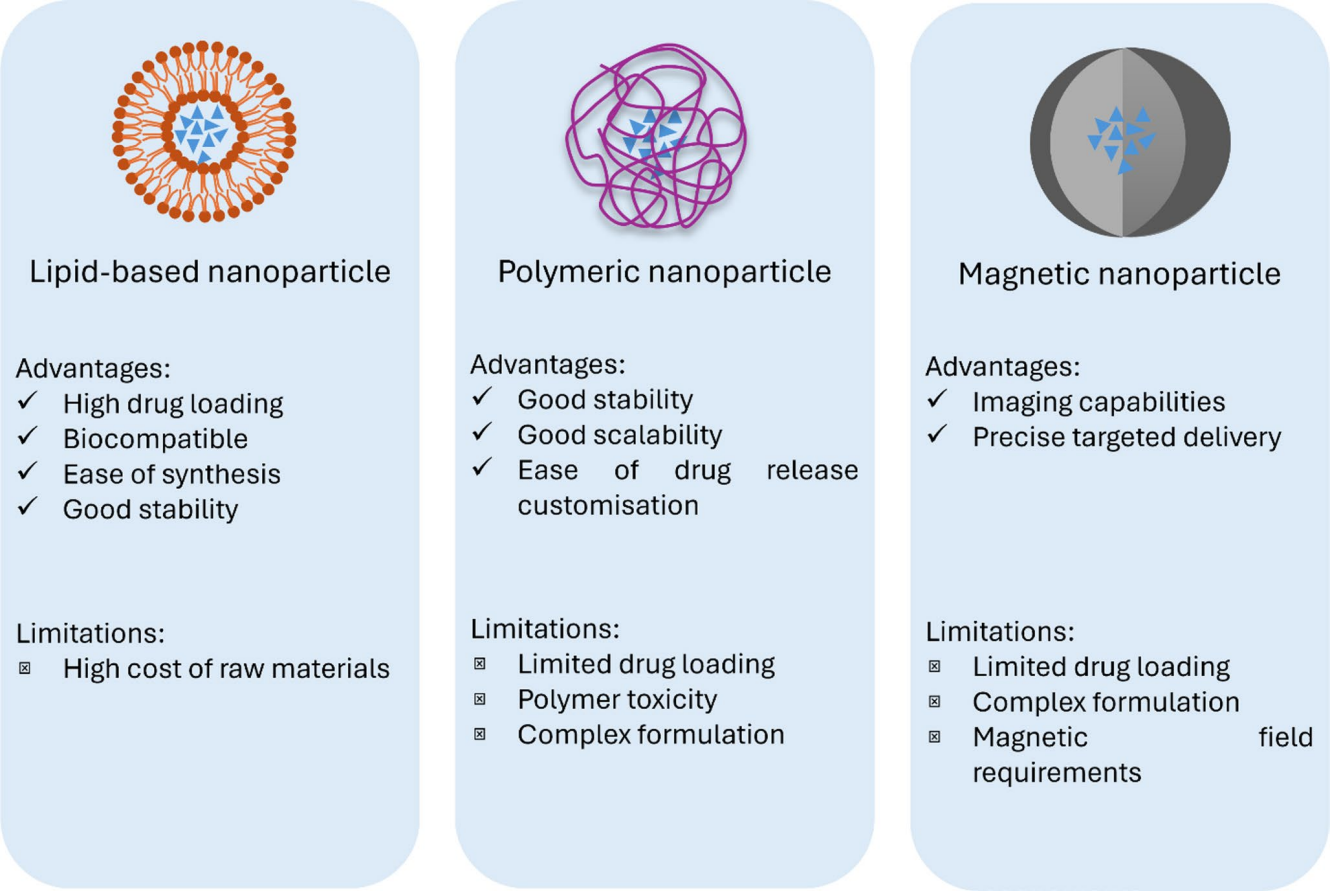
Comparison between different types of nanoparticles specifically used for the modification of ASMs

Nanocarriers have proven to provide a novel platform for the delivery of drugs, especially in controlled and site-specific drug delivery. These nanoscale carriers, typically 1 to 1000 nanometers, are designed to encapsulate and protect therapeutic agents, enhancing their stability, solubility, bioavailability, and targeting capabilities. In delivering ASMs to patients experiencing SE, the need for rapid and targeted delivery becomes particularly pronounced to arrest the seizure activity promptly [1] (Fig. 2).

### Lipid-based nanosystems

The utilization of lipid-based nanosystems has emerged as a promising strategy due to their high biocompatibility, which creates an optimal environment for drug encapsulation and precise targeting at therapeutic concentrations [22]. Lipid-based nanoparticles (LNPs) effectively address the solubility challenges associated with many ASMs, primarily due to their lipophilicity. By encapsulating lipophilic drugs within a lipid matrix, LNPs enhance their dispersion and absorption in biological systems [23]. This reduction in particle size and increase in surface area significantly improve the bioavailability of poorly soluble compounds, facilitating more efficient drug delivery. For example, solid lipid nanoparticles (SLNs) have been shown to effectively encapsulate indomethacin, resulting in improved bioavailability, regardless of various administration route such as ocular, oral and transdermal, as compared to conventional formulations [24–26]. Furthermore, LNPs protect drugs from enzymatic degradation by creating a shield that preserves their integrity in aqueous environments, thereby enhancing stability. Nanostructured lipid carriers exemplify this capability, as they not only improve solubility but also provide controlled release profiles for sensitive molecules

In addition to enhancing solubility and bioavailability, lipid-based nanoparticles play a crucial role in safeguarding the therapeutic integrity of ASMs and facilitating sustained drug release. The lipid matrix can gradually degrade or allow slow diffusion of the drug into the surrounding environment, ensuring a steady release over time and delivering drugs at optimal rates to achieve therapeutic effects without causing neurotoxicity [27, 28]. This controlled release profile minimizes fluctuations in drug concentrations, reducing potential side effects and ensuring prolonged therapeutic effects. Moreover, the ability of lipid-based nanocarriers to facilitate direct drug targeting enhances the overall efficacy of ASMs, making them an attractive option for overcoming challenges associated with conventional administration methods [29–31]. This targeted approach ensures more efficient drug delivery to the brain, optimizing treatment outcomes in the management of status epilepticus, as demonstrated by this systematic review.

### Polymeric-based nanoparticles

The polymeric-based nanoparticles share several advantages with lipid-based nanoparticles, particularly in improving drug solubility and biocompatibility. Both nanosystems also offer the ability to tailor drug release profiles, ensuring sustained and controlled release of ASMs. The most notable advantage of these nanosystems as highlighted in this systematic review in the management of SE is versatile ability to tailor for targeting delivery to specific brain regions. Moreover, the polymeric-based nanoparticles generally exhibit superior stability during storage compared to lipid-based nanoparticles [32]. It was due to their highly structured and stable matrix that resists deformation as observed on rigid polymers such as PLGA and PEG. Certain process that polymeric nanoparticles went through such as PEGylation could improve colloidal stability by reducing particle-particle interactions and prevent aggregation, thus enhancing long-term stability [33–35]. For instance, diazepam-PLGA loaded nanoparticles demonstrated prolonged stability lasting up to 18 months [19].

Moreover, these nano-systems offers a versatile platform for the co-delivery of multiple ASMs, enabling combination therapy. As demonstrated by Karthikeyini et al. [17], this approach is optimizing therapeutic outcomes by simultaneously targeting multiple pathways involved in seizure control. As a result, the nanoformulation of ASMs not only improves the biopharmaceutical properties but also allows offers the potential for tailored therapeutic interventions. Formulating ASMs into nanoformulations is one of the most promising strategic approaches to overcome challenges related to solubility, bioavailability, stability and ultimately enhancing the overall efficacy of these critical vital ASMs in the management of SE.

### Magnetic nanoparticles

Magnetic nanoparticles (MNPs) have emerged as a novel and highly promising drug delivery platform in central nervous system (CNS) disorders. Their magnetic responsiveness allows for external field manipulation, providing unique opportunities for targeted delivery, improved brain penetration, and site-specific drug accumulation. These features are particularly advantageous in the treatment of status epilepticus (SE), where rapid and localized drug action is critical.

MNPs are generally composed of iron oxide cores such as magnetite (Fe₃O₄) or maghemite (γ-Fe₂O₃) that are stabilized with biocompatible polymers, surfactants, or functional coatings that enable drug loading and dispersion. In the study by Hemmat et al. [36], phenobarbital was incorporated into MNPs using an aqueous microtitration method with Pluronic F-68, β-cyclodextrin, and ferrous sulfate heptahydrate. Despite the relatively large particle size (581 nm), the formulation demonstrated immediate release, indicating potential utility for fast-acting seizure control. The presence of cyclodextrin may also enhance drug solubility and stabilize the formulation within the systemic circulation.

What distinguishes MNPs from other nanocarriers is their magnetically guided delivery capability. By applying an external magnetic field near the targeted brain region, MNPs can be concentrated locally, thereby enhancing brain uptake, minimizing off-target distribution, and reducing systemic toxicity [37]. This feature is particularly significant when delivering central nervous system depressants like barbiturates, which require precise dosing to avoid adverse effects such as respiratory depression. In addition, the magnetic field may enhance BBB permeability by promoting magnetic force-assisted endocytosis or increasing the retention time of nanoparticles near the endothelial interface [38]. Manipulation of MNPs with surface ligands or antibodies for dual-targeting strategies like combining magnetic navigation with molecular recognition have a great potential in enhancing therapeutic efficacy by enabling selective delivery to inflamed brain regions or receptor-specific sites that are often associated with seizure foci [39].

However, several challenges remain before clinical translation. Long-term biocompatibility, iron accumulation risks, and oxidative stress associated with free iron species are concerns that require further investigation [40, 41]. Additionally, magnetic field application in clinical settings necessitates equipment that is both safe for cranial use and effective at guiding particles through dense biological matrices such as brain parenchyma. Integration with mucoadhesive intranasal carriers or PEGylation strategies may further optimize MNP performance and reduce potential toxicity [33].

MNPs provide a highly controllable, rapid-acting, and non-invasive delivery platform that is well suited for acute interventions in SE. The promising outcomes demonstrated in preclinical studies, including immediate release, enhanced BBB penetration, and targeted brain accumulation, suggest that MNPs represent a cutting-edge approach to overcome the limitations of conventional ASMs.

### Size, charges, and stability of nanoformulations

The size of nanoparticles plays a critical role in determining a properties, behaviour, and application of nanosystems. As nanoparticles are reduced in size, their surface area per unit mass increases significantly, allowing for increased drug loading capacity. This enhanced surface area also allows for more interaction with surrounding environments, making them highly reactive and effective in various applications [42]. Their nano scale enables them to penetrate biological barriers and reach specific target sites in the body. This property of the nanosystems has been used to improve the therapeutic efficiency of medications by ensuring their efficient delivery to the intended locations with higher precision, thereby optimizing treatment outcomes [43]. Study done on phenytoin sodium loaded into a nano lipid carrier is shown to have a better uptake and rapid accumulation of the drugs in the brain than the free phenytoin sodium solution [14]. An optimal nanoparticles size suggested by our systematic review is within the range of less than 250 d.nm and may be considered as favourable for targeted drug delivery [44]. However, it should be noted that the size itself is not the sole determinant of a nanosystem efficiency. The assumption that smaller particles are universally more favourable has been widely disproven. Studies have shown that smaller silver nanoparticles exhibit reduced colloidal stability due to extreme particle aggregation, hence, leading to loss of biological activity [45]. Additionally, greater toxicity towards the tested cell lines has been observed by the small sized nanoparticles [46]. These may be likely due to the accumulation of the particles at the targeted sites, leading to unwanted adverse effects.

Besides size, other physicochemical properties of nanoparticles, such as zeta potential, are often determined. Zeta potential study serves as a ‘tool’ to measure the stability of a colloidal system. In most cases, a stable system usually has charges of more than − 30 or + 30 mV as the charges between particles are theoretically strong enough to repel each other, reducing the chances of clumping or aggregation in the system [45]. This enhanced stability can contribute to maintaining the integrity of drug-loaded nanoparticles during storage and transportation, ensuring the delivery system remains effective until administration. On the other hand, the charges of the nanosystem could be a determinant factor in effectively delivering the drugs to their intended site, as most biological materials such as cell membranes, proteins, and mucosal surfaces also have charges [47]. With appropriate zeta potential values, nanoparticles can interact with target cells or tissue, enhancing cellular uptake and drug delivery efficiency, facilitating the adsorption of biomolecules onto nanoparticle surfaces, and further modulating drug release and biodistribution [48]. In delivering drugs into the BBB, where the interest mainly lies, nanoparticles with positive zeta potential are highly advantageous since the BBB cell membrane is negatively charged. This could lead to greater electrostatic interaction and more efficient drug transportation [49]. While measuring zeta potential may not be mandatory for all nanomedicine applications in seizure management, which has been demonstrated by small parts of the selected literature, it can provide valuable insights into nanoparticle behaviour, stability, and cellular interactions.

Pharmaceutical stability testing is a major investigation that studies the changes in the quality of any drug product with respect to time under the influence of environmental factors, such as temperature, humidity, and light. Stability testing is generally recommended during the product development of new drugs to establish a shelf-life for the drug product and to recommend a suitable storage condition, including temperature and humidity requirements [50]. For all new drug products, including nanomedicines, stability testing should include testing all parameters susceptible to change during transportation and storage and likely to influence the safety, efficacy, and quality of these products. One of the main highlights of using nanoparticles as a drug carrier is its ability to improve the stability of the drugs. Most of the time, ASMs are conventionally freshly prepared in a suspension, particularly for parenteral administration. They may face stability challenges due to chemical degradation as the drug particles are dispersed in a liquid medium and face changes in pH, oxidation, and hydrolysis [51]. Physical stability can also be compromised by factors such as temperature fluctuations, agitation during handling or storage, and exposure to light. Suspensions, particularly those containing aqueous components, may be susceptible to microbial contamination [52]. All these factors always compromise drug potency and efficacy. Nanoparticles can help overcome these stability issues by encapsulating ASMs acting as a protective carrier that safeguard the drug from degradation thus aiding in preserving the chemical integrity and stability of the drug, and hence extending its shelf life [7]. The small size and high surface area-to-volume ratio of nanoparticles facilitate uniform dispersion of the drug molecules, hence minimizing aggregation and precipitation, which are common causes of instability [53]. Although not all studies included in this systematic review reported stability studies, but the studies which reported stability studies demonstrated significant improvement in the stability of ASMs formulated into nanoparticle-based systems.

### Drug release behaviour

Investigations into drug release behaviour play a pivotal role in optimizing drug performance, particularly in the context of nanoparticle applications for nanomedicine. This aspect is intricately linked to formulation development, drug stability, and therapeutic efficacy. It is important to acknowledge that distinct release profiles are necessary for diverse epilepsy management strategies and ASMs. For instance, the utilization of an extended-release ASM formulation allows for a prolonged dosing interval, concurrently minimizing serum drug level fluctuations attributable to prolonged drug absorption, a particularly pertinent consideration for drugs with short half-lives, such as lorazepam, where maintaining stable therapeutic levels can significantly enhance seizure control [54].

Conversely, an immediate-release drug profile is critical in the management of SE, where rapid intervention is necessary to prevent neuronal damage. Notably, most literature consistently reports an immediate initial burst of drugs followed by a prolonged release period [12, 13, 15, 16, 18–20, 55]. A different study has shown that silica xerogel/polymer core shell nanoparticles can achieve a controlled two-stage drug release profile, where the first stage involves a rapid release of approximately 45% within the first day, followed by a more gradual release over the subsequent days [56]The versatility shown by different materials to synthesise nanoparticles in tailoring the release profile of drugs is advantageous in managing acute conditions like SE.

Benzodiazepines, which are commonly used as the first-line therapy for the management of SE, are administered in an immediate-release dosage form intravenously, allowing for rapid absorption and distribution, leading to a prompt response. They act quickly to enhance the inhibitory effects of GABA neurotransmission. Even intramuscular benzodiazepines that have an immediate release profile also have similar effectiveness as an intravenous dosage form for the treatment of SE in the prehospital setting, which has been demonstrated by Silbergleit et al. [57]. Clinical guidelines also recommend the use of immediate-release benzodiazepines for the initial treatment of SE [58]. While the aforementioned immediate-release drugs are beneficial for rapid seizure control, they may not provide sustained seizure suppression. However, additional ASMs with longer durations of action may be required for continued seizure management following the initial treatment with immediate-release agents [59]. The design of lipid-based and polymeric nanoparticles as what have been the main spotlight of most ASMs nanoformulations in this review allows for tailored release kinetics through adjustments in their compositions and structure. By modifying parameters such as particle size and polymeric concentration, researchers can influence the rate of drug diffusion and matrix degradation, thereby fine-tuning the release profiles to meet specific therapeutic needs [60, 61].

The immediate release dosage forms is characterized by typically releasing 70–85% or more of the ASMs within a specified time frame, often 30 min to 1 h, to ensure rapid onset of action [62]. While a majority of formulated compounds exhibit an initial burst release profile, which is advantageous for managing SE, a critical consideration arises regarding the adequacy of the dosage in halting seizures, given that the released dosage versus time often falls short of approximately 70% threshold. The initial dosage for the nanoformulated drugs varied widely among reviewed literature of this systematic review, making it difficult to determine the exact release percentage that may be needed for effective SE management. This variability may arise due multiple factors affecting drug release kinetics, including the type of nanoformulations, the carrier used for formulating nanoparticles and the physicochemical properties. In studies that involved animal models for biodistribution and pharmacokinetic study, all results showed a significantly higher brain uptake of the nanoformulated ASMs compared to the free ASMs suspension. In vivo, drug release kinetic assay, which more closely mimics the physiological conditions, may provide valuable insights into the drug behavior in the body, including its absorption, distribution, metabolism, and excretion [63]. For most of the nanoformulated ASMs, the drug release kinetics were best described by the Korsmeyer-peppas model following Fickian diffusion, highlighting the ability of the nanoparticles molecules to have a high permeability rate, particularly across the BBB. Nanoparticles with Fickian diffusion properties can exploit concentration gradients to passively diffuse across the BBB and reach the brain parenchyma, where they can exert their therapeutic effects [5]. The fact that the integrity of the BBB may be compromised during epilepsy due to inflammation, oxidative stress, and changes in the tight junctions that line the cerebral capillaries could also play a role in aiding the penetration and accumulation of nanoparticle drug molecules at sites of neuronal hyperexcitability associated with seizures [64].

Various in vivo [18, 20, 21, 55] and in vitro [13–16, 19, 55] assays, ranging from simple dialysis tube experiments to comprehensive investigations involving animal models, have been extensively employed to assess the release kinetics and pharmacokinetic behaviour of these nanoformulated ASMs. While each technique presents distinct advantages and limitations, integrating both approaches is often preferred to achieve a thorough understanding of drug release kinetics and the behavior of nanoformulated ASMs. However, while these release kinetic studies provide valuable data, the efficacy of these nanoformulated ASMs still require further evaluation through pharmacodynamic studies to elucidate their effectiveness of nanoformulated ASMs in terminating seizures, particularly in patients with SE.

### Route of administration

**Fig. 3:**
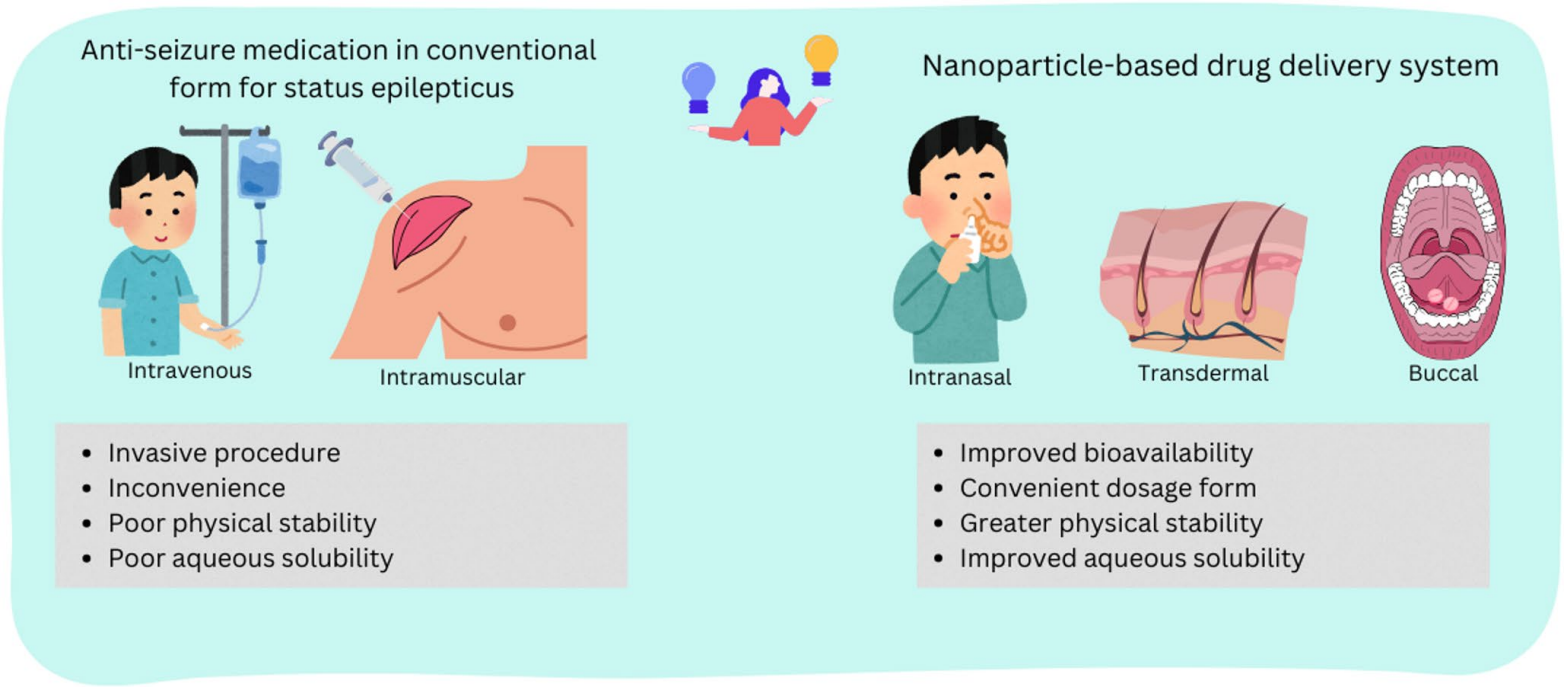
Differences between different types of administration route of conventional dosage form and nanoparticle-based delivery system of ASMs

Another critical consideration as listed in the data is the administration route of these ASM nanoformulations. The conventional and first line route for managing SE is parenteral route, primarily due to its fast onset of action. However, this route presents challenges related to invasiveness and the inconvenience it can cause to both the patients and healthcare staff [65]. The rectal route is another alternative but it also faces a significant barrier due strong public opinion in its inconvenience and discomfort it may cause to the patients and healthcare staff [66] (Fig. 3).

One of the main objectives of the nanoformulations for ASMs is to achieve precise control over drug delivery, facilitating targeted site-specific delivery. This aim can be achieved by modifying ASM to possess the properties that enhance their ability to reach and act at the site-specific, that is the brain. This systematic review reported that more than half of the studies seleted intranasal delivery as the route of choice, given its proven advantages for targeting the brain [67–69]. The intranasal route for benzodiazepine delivery has exhibited advantages over alternative administration routes. For instance, intranasal midazolam demonstrated comparable effectiveness to rectal diazepam but offered advantages such as easier administration, rapid onset of action, and enhanced societal acceptance [70]. Similarly, intranasal lorazepam demonstrated to be equally effective as intravenous administration in children aged 6–14 years; however, the intranasal route was preferred due to improved patient compliance [71]. The recent FDA approval of intranasal benzodiazepine sprays, such as Nayzilam and Voltaco, underscores the growing potential of this route for rescue therapies in status epilepticus (SE).

However, the intranasal route also poses challenges, including limited access volume (maximum 150 µL per nostril), brief residence time due to anterior leakage or posterior drainage, and the presence of degrading enzymes, and efflux transporters. The intranasal absorption is also variable and is influenced by physical factors such as nasal injury or pathological conditions like polyps, the common cold, and allergies [72]. Moreover, the physicochemical attributes of a drug, such as its size, water solubility, and stability, plays critical role in determining the rate and extent of absorption through the nasal route. For instance, drugs characterized by low water solubility, such as benzodiazepines, typically display suboptimal nasal absorption due to the limited aqueous dose that the nasal cavity can accommodate. Generally, smaller molecules (< 300 Da) are more readily absorbed nasally compared to larger molecules [72].

Several formulation strategies have been employed to mitigate previously discussed challenges concerning intranasal delivery and enhance direct or indirect delivery to the brain, including the nanoformulation of ASMs. This systematic review highlights that nanoformulated ASMs consistently report significant improvements in managing SE in animal models. Additionally, alternative routes of administration, such as transdermal, have also shown improvements compared to the control group, which received unmodified ASMs. However, less than half of the selected literature in this review explores the activity of the modified ASMs against animal seizure models – while the rest of literatures are formulation focus and only done until pharmacokinetic and biodistribution of the nanoparticles.

## Limitations

Since nanomedicine technology is quite recent, few concerns have been raised regarding potential irritation and toxicity, especially upon intranasal administration. The usage of surfactants and cosolvents may have long-term effects on the nasal lining and peripheral cells. Despite advancements, maintaining stability over extended periods remains challenging for some nanoparticle formulations. Polymeric nanoparticles, while generally more stable during storage than lipid-based counterparts, may still encounter degradation issues over time, necessitating ongoing stability assessments.

While smaller particle size offers advantages such as increased surface area and enhanced drug loading capacity, they may also exhibit reduced colloidal stability and increased aggregation, leading to diminished biological activity and greater toxicity. Achieving the optimal balance between size and functionality presents a challenge in nanoparticle design. The relationship between nanoparticle size and therapeutic efficiency is complex, with conflicting findings in the literature. While some studies demonstrate improved drug uptake and accumulation with smaller nanoparticles, others suggest that size alone is not the sole determinant of nanosystem efficiency, highlighting the need for further investigation.

Achieving desired drug release profiles, particularly in epilepsy management, presents a challenge due to the diverse requirements of different ASMs. Determining the adequacy of drug release to manage seizure effectively is challenging, as the released dosage may fall short of desired levels in in vitro assays. Variability in initial dosage and release kinetics further complicates assessment, highlighting the need for standardized evaluation methods. Variations in the drug release behaviour in nanoformulated drugs make it difficult to establish universal criteria for seizure management. Factors such as carrier types and physicochemical properties significantly influence drug release behaviour, necessitating a tailored approach for each formulation.

On the other hand, the absence of pharmacodynamic studies in most of the reviewed literature is particularly striking. Given that the primary objectives of these studies revolve around managing SE, the lack of investigations utilising epilepsy animal models raises significant concerns regarding the translational relevance of the findings. Pharmacodynamic studies are essential for understanding how modifications to drug formulations influence their action at the site of action, particularly within the CNS. Moreover, the reliance on pharmacokinetic data alone does not provide a comprehensive picture on how these modified ASMs perform in dynamic biological systems.

## Future perspectives

The future perspective on the use of nanomedicine for epilepsy treatment, considering the identified limitations and existing gaps in the management of SE, necessitates a multifaceted approach. First, addressing concerns regarding potential irritation and toxicity from intranasal administration is crucial; future research should focus on developing biocompatible materials that minimize adverse effects on nasal tissues. Additionally, the challenge of maintaining stability in nanoparticle formulations requires ongoing assessments and the exploration of advanced stabilization techniques to enhance shelf life. On the other hand, a clinical-scale magnetic targeting systems for MNPs, and multifunctional surface modifications can be done to fully exploit the potential of this technology in SE therapy. Optimizing particle size is also essential, as smaller sizes can improve drug loading but may reduce colloidal stability and increase toxicity; thus, establishing an optimal size range that balances these factors will be critical. Furthermore, achieving consistent drug release profiles tailored to the diverse requirements of ASMs calls for standardized evaluation methods to understand how formulation components influence drug release kinetics. Importantly, the notable absence of pharmacodynamic studies in current literature must be addressed by prioritizing evaluations using relevant epilepsy animal models to assess how modifications affect drug action within the CNS. Additionally, significant gaps in the management of SE must be considered, including under-recognition of seizures, lack of standardized definitions and treatment protocols, and limited access to specialized care for many patients. Bridging these gaps through improved education for healthcare providers and enhanced diagnostic protocols is essential for advancing SE management. Finally, bridging the gap between laboratory findings and clinical applications is vital for realizing the potential of nanomedicine in epilepsy treatment, requiring collaborative efforts among researchers, clinicians, and regulatory bodies to facilitate the transition from preclinical studies to clinical trials. By tackling these challenges through targeted research initiatives and addressing existing management gaps, nanomedicine could significantly enhance epilepsy management and provide new hope for affected individuals.

## Conclusion

Utilizing nanocarriers to deliver ASM, particularly in managing SE, is promising and innovative. Nanoformulations offer solutions to challenges such as poor water solubility, limited bioavailability, and stability issues associated with many ASM. By encapsulating ASM within nanoparticles, their solubility, bioavailability, and stability can be enhanced, leading to improved therapeutic outcomes. This systematic review has highlighted the effectiveness of lipid-based and polymeric nanoparticles in addressing the specific challenges associated with ASM and have a great potential in the management of SE. Future research should focus on refining nanoformulations to enhance efficacy and safety profiles, establishing standardized experimental design and evaluation protocols, and conducting robust clinical trials to validate nanoformulated ASM therapies in real-world settings. By translating preclinical findings into clinically viable therapies, nanoformulations hold significant promise in advancing the management of epilepsy, particularly in cases of status epilepticus.

## Data Availability

The datasets and materials used in this study are available from the corresponding author upon reasonable request.
